# Fuzzy optimal control of multilayer coverage based on radon exhalation dynamics in uranium tailings

**DOI:** 10.1038/s41598-023-31518-7

**Published:** 2023-03-17

**Authors:** Meirong Zhang, Jianyong Dai

**Affiliations:** 1 School of Resource Environment and Safety Engineering, University of South China, Hengyang, 421001 Hunan China; 2 Key Laboratory of Emergency Safety Technology and Equipment of Nuclear Facilities in Hunan Province, Hengyang, 421001 Hunan China

**Keywords:** Engineering, Materials science, Mathematics and computing

## Abstract

Radon exhalation from uranium tailings has seriously affected environmental safety and human health. Many uncertain parameters, such as diffusion coefficient, porosity, percolation rate, material particle size, etc., are related to the diffusion and migration of radon. Moreover, cover materials, cover layers, and cover thickness are the main instruments to control radon exhalation, and the radon reduction effect of single-layer mulching is often inferior to that of the multilayer. Hence, achieving radon control with multilayer coverage under uncertain environment is an urgent problem that must be solved in the area of nuclear safety and radiation environment. In an attempt to address the issue, a dynamic model of radon exhalation with multilayer coverage is constructed using radon percolation-diffusion migration equation, and triangular membership functions inscribe the model parameters; the objective functions of the left and right equations of the model are constructed, and their extreme value intervals are obtained using immunogenetic algorithm. Then, subject to the total cost and thickness of multilayer covering materials, the fuzzy objective and constraint models of radon exhalation are constructed, and the fuzzy aggregation function is reconstructed according to the importance of the fuzzy objective and constraint models, where ultimately, the optimal radon control decision by swarm intelligence algorithm under different possibility levels and importance conditions can be obtained. An example is then used to validate the effectiveness of the radon exhalation model, and to demonstrate that fuzzy optimization provides a database of decision-making schemes regarding multilayer coverage, and guidance for optimal control and flexible construction management.

## Introduction

Radionuclides not only diffuse in gaseous form in uranium tailings but also penetrate and migrate horizontally and vertically in the soil, causing serious radioactive pollution to soil, surface water, and groundwater, threatening the living environment of human beings, and restricting the sustainable development of the economy and society. Covering treatment is one of the most effective measures to reduce radon emissions from uranium tailings. It is covered with a soil layer, reduces the radon diffusion coefficient, increases the diffusion and residence time of free radon in the covering material, and ensures that radon decays in the covering layer before entering the atmosphere through the hysteresis effect, thus reducing radon emission. The migration of radon is controlled by many factors, and relevant examples have proven that multilayer coverage is the most effective way to reduce radon release from radioactive solid waste. Covering materials mainly involve many factors, such as the environment, cost, number and thickness of covering layers, construction safety, etc. The soil radon migration model reveals the migration speed of radon in different covering layers, and quantitatively explains the cause of abnormal radon in fault soil^1^. Three overburden physical models explain the asynchrony between surface radon anomalies and fault zones such as fissures, fissure system-fault zones, and heterogeneity. The thickness of the cover layer and soil structure properties directly affect the abnormal intensity and abnormal form of radon^2^. Weathering and alteration cause radionuclides to migrate to the surface of particles, and rock cracks are conducive to radon exhalation, while smaller particles, increased specific surface area, and increased rock inner surface area and porosity enrich and escape uranium and radium, thereby promoting radon exhalation from rock^3^. The inhibition of the radon exhalation rate shows that adding a zeolite layer to the cover material is more effective than adding a gypsum powder layer or fly ash layer in uranium tailings ponds^4–7^. The radon concentration exhalation is directly affected by parameters such as diffusion coefficient, convection velocity, overburden thickness, and ore body width^8^. In the meantime, radon control is a complex engineering system with multilayer coverage^9–11^. Because model parameters are uncertainty, they cannot describe dynamic models by the classical deterministic method, and can better solve dynamics modeling problems by membership function^12–14^. Therefore, it is particularly important for the optimal control of radon exhalation by fuzzy numbers in multilayer coverage under an uncertain environment.

Fuzzy model optimization is generally solved by converting it into a deterministic model, but some fuzzy models cannot be converted, otherwise, the inherent properties of the fuzzy model will be changed, and cannot be satisfied. In a fuzzy-stochastic environment, Kar R S used interval arithmetic technique to transform interval objective function into equivalent deterministic multi-objective problem. Stochastic and fuzzy-stochastic problems and their significant features were illustrated by numerical examples^15^. Intuitive fuzzy numbers are used to efficiently solve multi-objective non-linear optimization problems with unknown parameters, but every real-world problem cannot be rationalized and described using linear functions, hence efficient solution approaches, such as Zimmermann's technique, the maximum additive operator technique, and the γ-operator technique, have been enhanced by establishing nonlinear membership functions^16^. As an attractive generalization of the intuitionistic fuzzy set (IFS), the q-rung orthopedic fuzzy set (q-ROFS) provides the decision makers (DMs) with a wide window for preference elicitation^17^. The fuzzy adaptive teaching learning-based optimization method, which Cheng et al. proposed, employed three measures from the search space, namely, quality measures, diversification measures, and intensification measures, and was a newly updated teaching learning-based optimization algorithm^18^. Wu et al. provided the piecewise linear fuzzy geometric mean (PLFGM) method to increase the precision and effectiveness of predicting the fuzzy priority of criteria^19^. A method to calculate risk using the breadth of the interval uncertainty was also provided by Weldon A et al., along with tight limitations on the solutions of probabilistic optimization^20^. Lei et al. established a sequential optimization and reliability assessment (SORA) approach for multidisciplinary systems under hybrid interval and fuzzy uncertainties, the first-order Taylor expansion method, and the interval vertex method, both of which were developed using numerical and engineering methods. This is done to decouple the reliability analysis from the deterministic multidisciplinary design optimization (MDO)^21^. Therefore, it can be used to construct target and constraint extreme value intervals for radon exhalation models, reconstruct the fuzzy target and fuzzy constraint membership function by the membership function and swarm intelligence algorithm, and then construct a fuzzy integrated membership function according to the importance degrees of the target and constraint.

The fuzzy aggregate function model does not necessarily have derivatives and continuity, and there may be multi-modal functions. The classical optimization algorithm is a point search, and it is difficult to obtain the global optimal solution, while the swarm intelligence algorithm is a swarm search algorithm that does not require derivative information, and can find the global optimal solution^12,22,23^. Li gave modeling freedom, an easing of limitations, and permits the approach to look for a workable solution. To find the optimal compromise between all the objectives, the obtained fuzzy multi-objective optimization problem was handled using the non-dominated sorting genetic algorithm-II, which is renowned for its global searching skills^24^. Wang proposed a fuzzy simplified swarm optimization technique (fSSO) to handle the multi-objective optimization problem involving energy consumption, cost, and signal transmission quantity of the transmission process in WSNs under uncertainty^25^. Failure mode and effects analysis (FMEA) was used, and it did so by merging the multi-criteria technique Potentially All Pairwise RanKings of all conceivable Alternatives with an intuitionistic fuzzy hybrid weighted Euclidean distance operator (PAPRIKA)^26^. To forecast the productivity of a factory, Chiu et al. presented an interval fuzzy number (IFN)-based mixed binary quadratic programming-ordered weighted average (OWA) technique, with the results showing that it was more accurate than several other methods in terms of a variety of metrics^27^. Kim and Jung suggested α-level estimation algorithm for ridge fuzzy regression modeling. By including α-levels in the estimation process, a fuzzy ridge estimator that is independent of the distance between fuzzy numbers is created^28^. To manage water resources, Wang et al. incorporated a number of risk control constraints, such as water availability, maximum permitted penalties, and allowable benefit violation limitations, into a fuzzy border interval two-stage stochastic programming framework. This framework addresses the recourse action to reduce penalties based on the interacting effects of various risk control measures, further creating optimum water allocation options, and directing water resource management^29^. Therefore, it can obtain the optimal solution of radon exhalation models under uncertain environments by a membership function and swarm intelligence algorithm, which carries out multi-stage optimal programming.

In summary, the radon exhalation dynamics are not only related to its state but also related to the control variables. Given that radon exhalation parameters are uncertain, it constructs radon exhalation fuzzy targets and fuzzy constraint equations by the membership function, and sets up fuzzy integration functions with different probability levels and different importance, gets the database of optimal decision-making construction scheme by swarm intelligence algorithm, which provides the basis of optimal control decisions for multilayered coverage in uranium tailings piles.

## Dynamics of radon exhalation from multilayer coverage of uranium tailings

### Migration dynamics of radioactive contaminants

Controlling radon exhalation by covering is an important step to achieve environmental management of uranium tailing piles. The effect of single-layer coverage is often not as effective as double-layer coverage for radon reduction when covered with the same thickness of coverage material. Considering the radon reduction effect and cost consumption and other factors, multilayer coverage can be applied to achieve the optimal radon reduction effect. The migration of radioactive contaminants undergoes decay reactions, which can be described by Eq. (1) or Eq. (2), and the analytical solution of Eq. (3) for the semi-infinite domain problem is obtained by derivation using the Laplace transform.

1 $$\frac{\partial C}{{\partial t}} = div\left( {DgradC} \right) - div\left( {uC} \right) + f\left( C \right)$$

2 $$\left\{ \begin{gathered} \frac{{\partial C\left( {x,t} \right)}}{\partial t} = D_{L} \frac{{\partial^{2} C\left( {x,t} \right)}}{{\partial x^{2} }} - u\frac{{\partial C\left( {x,t} \right)}}{\partial x} - \lambda C\left( {x,t} \right) \hfill \\ \left. {C\left( {x,t} \right)} \right|_{t = 0} = C_{0} \hfill \\ \end{gathered} \right.$$

3 $$\left\{ \begin{gathered} C\left( {x,t} \right) = \frac{{C_{0} }}{2}\left[ {\exp \left[ {\frac{{\left( {u - w} \right)x}}{{2D_{L} }}} \right]erfc\left( {\frac{x - wt}{{2\sqrt {D_{L} t} }}} \right) + \exp \left[ {\frac{{\left( {u + w} \right)x}}{{2D_{L} }}} \right]erfc\left( {\frac{x + wt}{{2\sqrt {D_{L} t} }}} \right)} \right] \hfill \\ w = \sqrt {u^{2} + 4\lambda D_{L} } \hfill \\ \end{gathered} \right.$$

where $c(x,t)$ is the pollutant concentration, ML^−3^; $D_{L}$ is the dispersion coefficient, L^2^T^−1^; $\lambda$ is the reaction constant; u is the actual flow rate, LT^−1^.

### Two-layer coverage radon exhalation model

**Figure 1:**
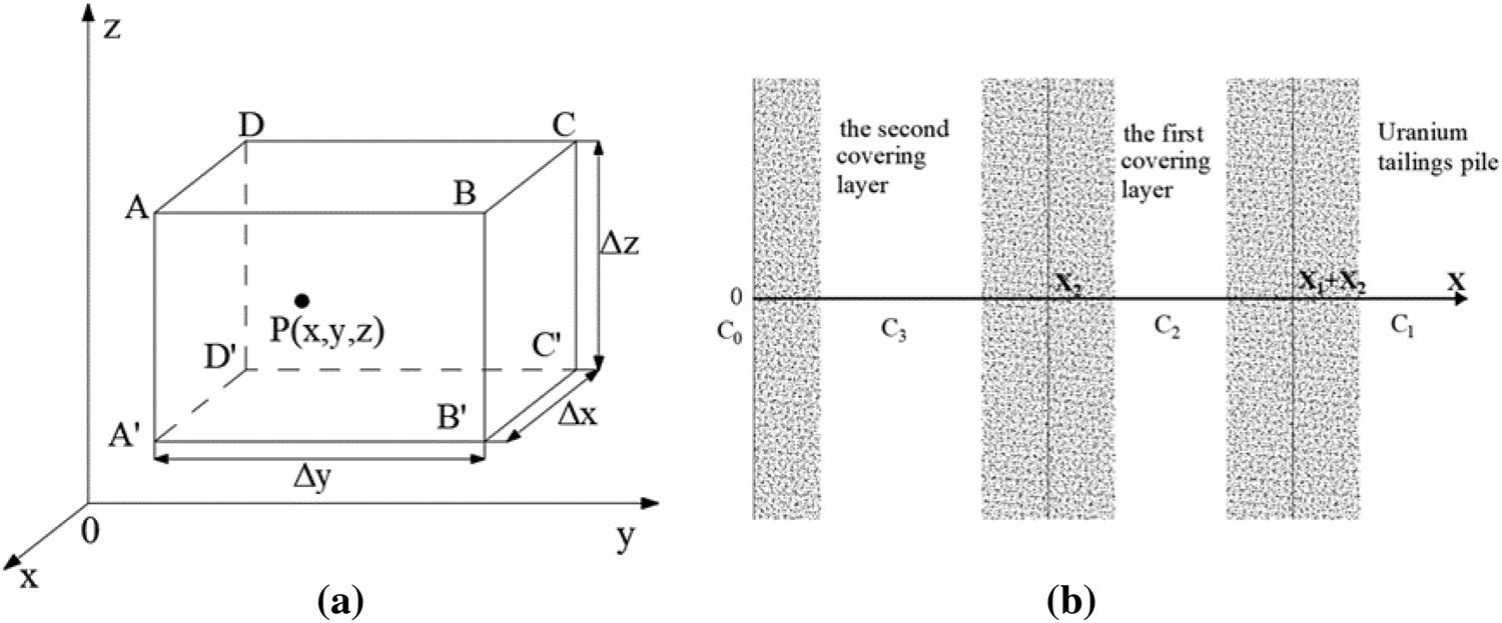
(**a**) Calculated coordinates of the element volume. (**b**) Calculated coordinates of the two-layer coverage model.

The differential equation of pollutants is the differential dynamics equation of convection and dispersion, infinitesimal parallelepiped with the p point is set as the center in the research field full of fluids, whose side lengths $D_{3}$,${{m^{2} } \mathord{\left/ {\vphantom {{m^{2} } s}} \right. \kern-0pt} s}$, and $C_{3}$ are taken as the equilibrium unit body (Fig. 1a).

The variation law of radon migration in uranium tailings is affected by many factors, such as the cover material parameters, cover thickness, cover level, construction sequence and construction cost. Single-layer coverage is often less effective than double-layer or multi-layer coverage, so different coverage levels and thicknesses such as $x_{1}$ and $x_{2}$ can be designed to control the radon concentration, such as $c_{2}$ and $c_{3}$(Fig. 1b).

For the case that the cover layer does not contain radium-226, assuming that the covered uranium tailings pile is a semi-infinite homogeneous porous jet medium, the equations of radon concentration distribution in the tailings pile under a steady-state are

$$X_{1} + X_{2} \le X$$

4 $$D_{1} \frac{{d^{2} C_{1} }}{{dx^{2} }} - v\frac{{dC_{1} }}{dx} - \lambda C_{1} + \alpha = 0$$

The distribution equations of radon concentration in the first cover layer under steady-state are

$$X_{1} < X \le X_{1} + X_{2}$$

5 $$D_{2} \frac{{d^{2} C_{2} }}{{dx^{2} }} - v\frac{{dC_{2} }}{dx} - \lambda C_{2} = 0$$

The distribution equations of radon concentration in the second cover layer under steady-state are

$$X \le X_{1}$$

6 $$D_{3} \frac{{d^{2} C_{3} }}{{dx^{2} }} - v\frac{{dC_{3} }}{dx} - \lambda C_{3} = 0$$

According to boundary conditions

$$x \to \infty ,\;C_{1} = \frac{a}{\lambda }$$

$$x = 0,C_{3} = \eta_{3} C_{0} ;$$

$$x = x_{1} ,\frac{{C_{3} }}{{\eta_{3} }} = \frac{{C_{2} }}{{\eta_{2} }}and\frac{{dC_{3} }}{dx} = \frac{{dC_{2} }}{dx};$$

$$x = x_{1} + x_{2} ,\frac{{C_{1} }}{{\eta_{1} }} = \frac{{C_{2} }}{{\eta_{2} }}and\frac{{dC_{1} }}{dx} = \frac{{dC_{2} }}{dx}$$

Equations (4), (5), and (6) are solved respectively to obtain the following results.

7 $$C_{1} = b_{1} e^{{p_{1} \left( {x - x_{1} - x_{2} } \right)}} + \frac{\alpha }{\lambda },{{Bq} \mathord{\left/ {\vphantom {{Bq} {m^{3} }}} \right. \kern-0pt} {m^{3} }}$$

8 $$C_{2} = a_{2} e^{{w_{2} (x - x_{1} )}} + b_{2} e^{{p_{2} (x - x_{1} )}} ,{{Bq} \mathord{\left/ {\vphantom {{Bq} {m^{3} }}} \right. \kern-0pt} {m^{3} }}$$

9 $$C_{3} = a_{3} \left( {e^{{w_{3} x}} - e^{{p_{3} x}} } \right) + \eta_{3} C_{0} e^{{p_{3} x}} ,{{Bq} \mathord{\left/ {\vphantom {{Bq} {m^{3} }}} \right. \kern-0pt} {m^{3} }}$$

where

10 $$w_{i} = \frac{{v + \sqrt {v^{2} + 4\lambda D{}_{i}} }}{{2D_{i} }},m^{ - 1} ,(i = 1,2,3)$$

11 $$p_{i} = \frac{{v - \sqrt {v^{2} + 4\lambda D_{i} } }}{{2D_{i} }},m^{ - 1} ,(i = 1,2,3)$$

12 $$a_{3} = \frac{{w_{2} p_{1} \frac{\alpha }{\lambda } - p_{1} p_{2} \frac{\alpha }{\lambda } - \left[ {L_{2} \left( {w_{2} n - p_{2} m} \right) - \left( {n - m} \right)p_{3} } \right]\eta_{3} C_{0} e^{{p_{3} x_{1} }} }}{{L_{2} \left( {e^{{w_{3} x_{1} }} - e^{{p_{3} x_{1} }} } \right)\left( {w_{2} n - p_{2} m} \right) - \left( {n - m} \right)\left( {w_{3} e^{{w_{3} x_{1} }} - p_{3} e^{{p_{3} x_{1} }} } \right)}}$$

where $x_{{}}$ is the total thickness of the cover, m. $x_{1}$ is the thickness of the second cover, m. $x_{2}$ is the thickness of the first cover, m. $\lambda$ is the decay constant of radon. $D_{1}$ is the diffusion coefficient of radon in tailings pile, m^2^/s. $C_{1}$ is the concentration of radon in the tailings, Bq/m^3^. $\eta_{1}$ is the porosity of the uranium tailings. $D_{2}$ is the diffusion coefficient of radon in the covering layer, m^2^/s. $C_{2}$ is the concentration of radon in the first covering layer, Bq/m^3^. $\eta_{2}$ is the porosity of the first covering material. $D_{3}$ is the diffusion coefficient of radon in the second cover layer, m^2^/s. $C_{3}$ is the concentration of radon in the second cover layer, Bq/m^3^. $\eta_{3}$ the porosity of the second cover material.

The radon exhalation rate on the surface of the double-layer covering system is obtained as

13 $$\begin{gathered} J = D_{2} \frac{{w_{2} p_{1} \frac{\alpha }{\lambda } - p_{1} p_{2} \frac{\alpha }{\lambda } - \left[ {L_{2} \left( {w_{2} n - p_{2} m} \right) - \left( {n - m} \right)p_{3} } \right]\eta_{3} C_{0} e^{{p_{3} x_{1} }} }}{{L_{2} \left( {e^{{w_{3} x_{1} }} - e^{{p_{3} x_{1} }} } \right)\left( {w_{2} n - p_{2} m} \right) - \left( {n - m} \right)\left( {w_{3} e^{{w_{3} x_{1} }} - p_{3} e^{{p_{3} x_{1} }} } \right)}}\left( {w_{3} - p_{3} } \right) \hfill \\ + D_{3} p_{3} \eta_{3} C_{0} - v\eta_{3} C_{0} \hfill \\ \end{gathered}$$

where, parameters m, n, L_1_, and L_2_ are shown in formula (14)

14 $$\left\{ \begin{gathered} m = \left( {p_{1} L_{{_{1} }} - w_{2} } \right)e^{{w_{2} x_{2} }} \hfill \\ n = \left( {p_{1} L_{{_{1} }} - p_{2} } \right)e^{{p_{2} x_{2} }} \hfill \\ L_{1} = \frac{{\eta_{1} }}{{\eta_{2} }} \hfill \\ L_{2} = \frac{{\eta_{2} }}{{\eta_{3} }} \hfill \\ \end{gathered} \right.$$

The control model of radon exhalation of two-layer covering of uranium tailings pile is

15 $$\begin{gathered} \min J = - 4\alpha \sqrt {\frac{{\lambda D_{3} }}{{D_{1} D_{2} }}} \cdot \left[ {\frac{{\eta_{2} }}{{\eta_{3} }}\sqrt {\frac{\lambda }{{D_{2} }}} } \right.\left( {e^{{\sqrt {\frac{\lambda }{{D_{3} }}} x_{1} }} - e^{{ - \sqrt {\frac{\lambda }{{D_{3} }}} x_{1} }} } \right) \hfill \\ \left[ {\left( {\sqrt {\frac{\lambda }{{D_{2} }}} - \frac{{\eta_{1} }}{{\eta_{2} }}\sqrt {\frac{\lambda }{{D_{1} }}} } \right)e^{{ - \sqrt {\frac{\lambda }{{D_{2} }}} x_{2} }} - \left( {\sqrt {\frac{\lambda }{{D_{2} }}} + \frac{{\eta_{1} }}{{\eta_{2} }}\sqrt {\frac{\lambda }{{D_{1} }}} } \right)e^{{\sqrt {\frac{\lambda }{{D_{2} }}} x_{2} }} } \right] \hfill \\ - \left. {{\kern 1pt} {\kern 1pt} {\kern 1pt} \sqrt {\frac{\lambda }{{D_{3} }}} \left( {e^{{\sqrt {\frac{\lambda }{{D_{3} }}} x_{1} }} + e^{{ - \sqrt {\frac{\lambda }{{D_{3} }}} x_{1} }} } \right)\left[ {\left( {\sqrt {\frac{\lambda }{{D_{2} }}} - \frac{{\eta_{1} }}{{\eta_{2} }}\sqrt {\frac{\lambda }{{D_{1} }}} } \right)e^{{ - \sqrt {\frac{\lambda }{{D_{2} }}} x_{2} }} + \left( {\sqrt {\frac{\lambda }{{D_{2} }}} + \frac{{\eta_{1} }}{{\eta_{2} }}\sqrt {\frac{\lambda }{{D_{1} }}} } \right)e^{{\sqrt {\frac{\lambda }{{D_{2} }}} x_{2} }} } \right]} \right]^{ - 1} \hfill \\ \end{gathered}$$

### Multilayer coverage radon exhalation model

For the case that the cover layer does not contain radium-226, the general equation of radon steady-state diffusion propagation in multilayer homogeneous porous media is

16 $$D_{i} \frac{{d^{2} C_{i} }}{{dx^{2} }} - v_{i} \frac{{dC_{i} }}{dx} - \lambda C_{i} = 0\left( {i = 1,2,...,n} \right)$$

Its general solution is

17 $$C_{i} (x) = ae^{{\left[ {\frac{{v + \sqrt {v^{2} + 4\lambda D_{i} } }}{{2D_{i} }}} \right]\left( {x - \sum\limits_{n = 1}^{i - 1} {x_{n} } } \right)}} + be^{{\left[ {\frac{{v - \sqrt {v^{2} + 4\lambda D_{i} } }}{{2D_{i} }}} \right]\left( {x - \sum\limits_{n = 1}^{i - 1} {x_{n} } } \right)}}$$

The radon exhalation rate on the surface of the overlay is J. The expression of radon exhalation from the multilayer overlay is as follows.

18 $$J = D_{n + 1} a_{n + 1} \left( {w_{n + 1} - p_{n + 1} } \right) + D_{n + 1} p_{n + 1} \eta_{n + 1} c_{0} - v\eta_{n + 1} c_{0}$$

where

19 $$\left\{ \begin{gathered} a_{n + 1} = f\left( {\eta ,v,D} \right) \hfill \\ w_{n + 1} = \frac{{v + \sqrt {v^{2} + 4\lambda D_{n + 1} } }}{{2D_{n + 1} }} \hfill \\ p_{n + 1} = \frac{{v - \sqrt {v^{2} + 4\lambda D_{n + 1} } }}{{2D_{n + 1} }} \hfill \\ \end{gathered} \right.$$

**Figure 2:**
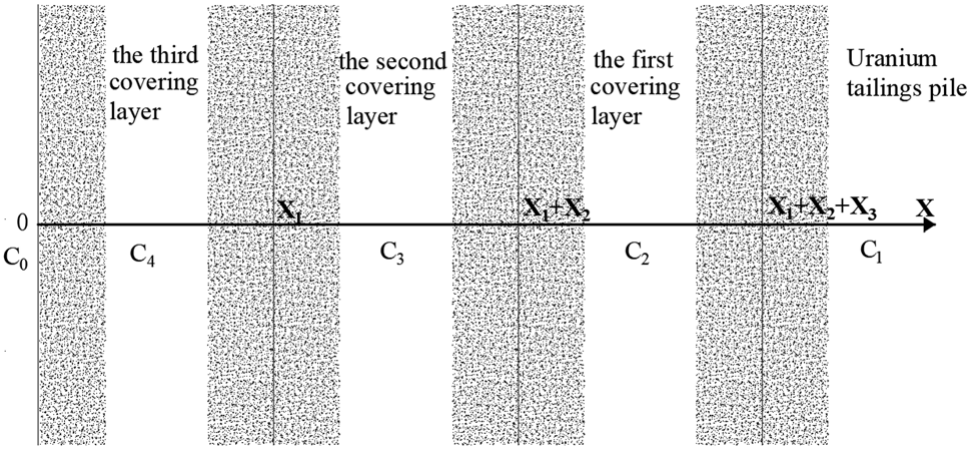
Calculated coordinates of the three-layer coverage model.

$c_{n}$ is the number of cover layers. $D_{n + 1}$ is the diffusion coefficient of radon in the nth cover layer. $\eta_{n + 1}$ is the porosity in the nth cover layer. Then the radon exhalation equation on the surface of the uranium tailings pile is covered by three layers (Fig. 2) as shown in Eq. (20).

20 $$\begin{gathered} {{J = - 4\alpha \lambda \left( {\sqrt {\frac{{D_{4} }}{{D_{1} D_{2} D_{3} }}} - \frac{1}{{D_{1} }}\sqrt {\frac{{D_{4} }}{{D_{2} }}} } \right)} \mathord{\left/ {\vphantom {{J = - 4\alpha \lambda \left( {\sqrt {\frac{{D_{4} }}{{D_{1} D_{2} D_{3} }}} - \frac{1}{{D_{1} }}\sqrt {\frac{{D_{4} }}{{D_{2} }}} } \right)} {\left[ {\left( {\frac{\lambda }{{\sqrt {D_{2} D_{3} } }} + \frac{{\eta_{2} \lambda }}{{\eta_{3} D_{2} }} - \frac{{\eta_{1} }}{{\eta_{2} }}\frac{\lambda }{{\sqrt {D_{1} D_{3} } }} - \frac{{\eta_{1} }}{{\eta_{3} }}\frac{\lambda }{{\sqrt {D_{1} D_{2} } }}} \right)e^{{ - \sqrt {\frac{\lambda }{{D_{2} }}} x_{1} - \sqrt {\frac{\lambda }{{D_{3} }}} x_{2} }} + } \right.}}} \right. \kern-0pt} {\left[ {\left( {\frac{\lambda }{{\sqrt {D_{2} D_{3} } }} + \frac{{\eta_{2} \lambda }}{{\eta_{3} D_{2} }} - \frac{{\eta_{1} }}{{\eta_{2} }}\frac{\lambda }{{\sqrt {D_{1} D_{3} } }} - \frac{{\eta_{1} }}{{\eta_{3} }}\frac{\lambda }{{\sqrt {D_{1} D_{2} } }}} \right)e^{{ - \sqrt {\frac{\lambda }{{D_{2} }}} x_{1} - \sqrt {\frac{\lambda }{{D_{3} }}} x_{2} }} + } \right.}} \hfill \\ {\kern 1pt} {\kern 1pt} {\kern 1pt} {\kern 1pt} {\kern 1pt} {\kern 1pt} {\kern 1pt} {\kern 1pt} {\kern 1pt} {\kern 1pt} {\kern 1pt} {\kern 1pt} {\kern 1pt} {\kern 1pt} {\kern 1pt} {\kern 1pt} {\kern 1pt} {\kern 1pt} {\kern 1pt} {\kern 1pt} \left. {\left( {\frac{\lambda }{{\sqrt {D_{2} D_{3} } }} - \frac{{\eta_{2} \lambda }}{{\eta_{3} D_{2} }} + \frac{{\eta_{1} }}{{\eta_{2} }}\frac{\lambda }{{\sqrt {D_{1} D_{3} } }} - \frac{{\eta_{1} }}{{\eta_{3} }}\frac{\lambda }{{\sqrt {D_{1} D_{2} } }}} \right)e^{{\sqrt {\frac{\lambda }{{D_{2} }}} x_{1} - \sqrt {\frac{\lambda }{{D_{3} }}} x_{2} }} } \right]\left[ {\left( {\frac{{\eta_{3} }}{{\eta_{4} }}\sqrt {\frac{\lambda }{{D_{3} }}} - \sqrt {\frac{\lambda }{{D_{4} }}} } \right)} \right.e^{{\sqrt {\frac{\lambda }{{D_{4} }}} x_{3} }} - \left( {\frac{{\eta_{3} }}{{\eta_{4} }}\sqrt {\frac{\lambda }{{D_{3} }}} } \right. \hfill \\ {\kern 1pt} {\kern 1pt} {\kern 1pt} {\kern 1pt} {\kern 1pt} {\kern 1pt} {\kern 1pt} {\kern 1pt} {\kern 1pt} {\kern 1pt} {\kern 1pt} {\kern 1pt} {\kern 1pt} {\kern 1pt} \left. {{\kern 1pt} {\kern 1pt} {\kern 1pt} {\kern 1pt} {\kern 1pt} \left. { + \sqrt {\frac{\lambda }{{D_{4} }}} } \right)e^{{ - \sqrt {\frac{\lambda }{{D_{4} }}} x_{3} }} } \right] + \left[ {\left( {\frac{\lambda }{{\sqrt {D_{2} D_{3} } }} - \frac{{\eta_{2} \lambda }}{{\eta_{3} D_{2} }} - \frac{{\eta_{1} }}{{\eta_{2} }}\frac{\lambda }{{\sqrt {D_{1} D_{3} } }} + \frac{{\eta_{1} }}{{\eta_{3} }}\frac{\lambda }{{\sqrt {D_{1} D_{2} } }}} \right)} \right.e^{{\sqrt {\frac{\lambda }{{D_{3} }}} x_{2} - \sqrt {\frac{\lambda }{{D_{2} }}} x_{1} }} + \left( {\frac{\lambda }{{\sqrt {D_{2} D_{3} } }} + \frac{{\eta_{2} \lambda }}{{\eta_{3} D_{2} }}} \right. \hfill \\ {\kern 1pt} {\kern 1pt} {\kern 1pt} {\kern 1pt} {\kern 1pt} {\kern 1pt} {\kern 1pt} {\kern 1pt} {\kern 1pt} {\kern 1pt} {\kern 1pt} {\kern 1pt} {\kern 1pt} {\kern 1pt} {\kern 1pt} {\kern 1pt} \left. {\left. { + \frac{{\eta_{1} }}{{\eta_{2} }}\frac{\lambda }{{\sqrt {D_{1} D_{3} } }} + \frac{{\eta_{1} }}{{\eta_{3} }}\frac{\lambda }{{\sqrt {D_{1} D_{2} } }}} \right)e^{{\sqrt {\frac{\lambda }{{D_{3} }}} x_{2} + \sqrt {\frac{\lambda }{{D_{2} }}} x_{1} }} } \right]\left[ {\left( {\frac{{\eta_{3} }}{{\eta_{4} }}\sqrt {\frac{\lambda }{{D_{3} }}} - \sqrt {\frac{\lambda }{{D_{4} }}} } \right)e^{{ - \sqrt {\frac{\lambda }{{D_{4} }}} x_{3} }} - \left( {\frac{{\eta_{3} }}{{\eta_{4} }}\sqrt {\frac{\lambda }{{D_{3} }}} + \sqrt {\frac{\lambda }{{D_{4} }}} } \right)e^{{\sqrt {\frac{\lambda }{{D_{4} }}} x_{3} }} } \right] \hfill \\ \end{gathered}$$

where $x_{1}$ is the thickness of the third cover, m. $x_{2}$ is the thickness of the second cover, m. x_3_ is the thickness of the first cover, m. $D_{4}$ is the diffusion coefficient of radon in the third cover layer, m^2^/s. $C_{4}$ is the concentration of radon in the third cover layer, Bq/m^3^. $\eta_{4}$ the porosity of the third cover material.

## Fuzzy optimization of radon exhalation from multilayer coverage of uranium tailings

### Fuzzy model of radon exhalation

For the case where the cover layer does not contain radium-226, the general equation of radon steady-state diffusion in the multilayer uniform porous medium is Eq. (21).

21 $$\tilde{D}_{i} \frac{{d^{2} \tilde{C}_{i} }}{{dx^{2} }} - \tilde{v}_{i} \frac{{d\tilde{C}_{i} }}{dx} - \lambda \tilde{C}_{i} = 0\left( {i = 1,2,...,n} \right)$$

Its general solution is

22 $$\tilde{C}_{i} (x) = \tilde{a}e^{{\left[ {\frac{{\tilde{v} + \sqrt {\tilde{v}^{2} + 4\lambda \tilde{D}_{i} } }}{{2\tilde{D}_{i} }}} \right]\left( {x - \sum\limits_{n = 1}^{i - 1} {x_{n} } } \right)}} + \tilde{b}e^{{\left[ {\frac{{\tilde{v} - \sqrt {\tilde{v}^{2} + 4\lambda \tilde{D}_{i} } }}{{2\tilde{D}_{i} }}} \right]\left( {x - \sum\limits_{n = 1}^{i - 1} {x_{n} } } \right)}}$$

Assuming that the radon exhalation rate on the surface of the coating layer is J, the radon exhalation of the multilayer covering is expressed as Eq. (23).

23 $$J = \tilde{D}_{n + 1} \tilde{a}_{n + 1} \left( {\tilde{w}_{n + 1} - \tilde{p}_{n + 1} } \right) + \tilde{D}_{n + 1} \tilde{p}_{n + 1} \tilde{\eta }_{n + 1} c_{0} - \tilde{v}\tilde{\eta }_{n + 1} c_{0}$$

Here,

24 $$\left( \begin{gathered} \tilde{a}_{n + 1} = f\left( {\tilde{\eta },\tilde{v},\tilde{D}} \right) \hfill \\ \tilde{w}_{n + 1} = \frac{{\tilde{v} + \sqrt {\tilde{v}^{2} + 4\lambda \tilde{D}_{n + 1} } }}{{2\tilde{D}_{n + 1} }} \hfill \\ \tilde{p}_{n + 1} = \frac{{\tilde{v} - \sqrt {\tilde{v}^{2} + 4\lambda \tilde{D}_{n + 1} } }}{{2\tilde{D}_{n + 1} }} \hfill \\ \end{gathered} \right.$$

$c_{n}$ is the number of cover layers. $\tilde{D}_{n + 1}$ is the fuzzy diffusion coefficient of radon in the nth cover layer. $\tilde{\eta }_{n + 1}$ is the fuzzy porosity in the nth cover layer. Then the fuzzy model of radon exhalation on the surface of the three-layer coverage is Eq. (25).

25 $$\begin{gathered} f\left( {\tilde{\beta },x_{1} ,x_{2} ,x_{3} } \right) \hfill \\ {{ = - 4\tilde{\alpha }\lambda \left( {\sqrt {\frac{{\tilde{D}_{4} }}{{\tilde{D}_{1} \tilde{D}_{2} \tilde{D}_{3} }}} - \frac{1}{{\tilde{D}_{1} }}\sqrt {\frac{{\tilde{D}_{4} }}{{\tilde{D}_{2} }}} } \right)} \mathord{\left/ {\vphantom {{ = - 4\tilde{\alpha }\lambda \left( {\sqrt {\frac{{\tilde{D}_{4} }}{{\tilde{D}_{1} \tilde{D}_{2} \tilde{D}_{3} }}} - \frac{1}{{\tilde{D}_{1} }}\sqrt {\frac{{\tilde{D}_{4} }}{{\tilde{D}_{2} }}} } \right)} {\left[ {\left( {\frac{\lambda }{{\sqrt {\tilde{D}_{2} \tilde{D}_{3} } }} + \frac{{\tilde{\eta }_{2} \lambda }}{{\tilde{\eta }_{3} D_{2} }} - \frac{{\tilde{\eta }_{1} }}{{\tilde{\eta }_{2} }}\frac{\lambda }{{\sqrt {\tilde{D}_{1} \tilde{D}_{3} } }} - \frac{{\tilde{\eta }_{1} }}{{\tilde{\eta }_{3} }}\frac{\lambda }{{\sqrt {\tilde{D}_{1} \tilde{D}_{2} } }}} \right)e^{{ - \sqrt {\frac{\lambda }{{\tilde{D}_{2} }}} x_{1} - \sqrt {\frac{\lambda }{{\tilde{D}_{3} }}} x_{2} }} } \right.}}} \right. \kern-0pt} {\left[ {\left( {\frac{\lambda }{{\sqrt {\tilde{D}_{2} \tilde{D}_{3} } }} + \frac{{\tilde{\eta }_{2} \lambda }}{{\tilde{\eta }_{3} D_{2} }} - \frac{{\tilde{\eta }_{1} }}{{\tilde{\eta }_{2} }}\frac{\lambda }{{\sqrt {\tilde{D}_{1} \tilde{D}_{3} } }} - \frac{{\tilde{\eta }_{1} }}{{\tilde{\eta }_{3} }}\frac{\lambda }{{\sqrt {\tilde{D}_{1} \tilde{D}_{2} } }}} \right)e^{{ - \sqrt {\frac{\lambda }{{\tilde{D}_{2} }}} x_{1} - \sqrt {\frac{\lambda }{{\tilde{D}_{3} }}} x_{2} }} } \right.}} \hfill \\ + \left. {\left( {\frac{\lambda }{{\sqrt {\tilde{D}_{2} \tilde{D}_{3} } }} - \frac{{\tilde{\eta }_{2} \lambda }}{{\tilde{\eta }_{3} \tilde{D}_{2} }} + \frac{{\tilde{\eta }_{1} }}{{\tilde{\eta }_{2} }}\frac{\lambda }{{\sqrt {\tilde{D}_{1} \tilde{D}_{3} } }} - \frac{{\tilde{\eta }_{1} }}{{\tilde{\eta }_{3} }}\frac{\lambda }{{\sqrt {\tilde{D}_{1} \tilde{D}_{2} } }}} \right)e^{{\sqrt {\frac{\lambda }{{\tilde{D}_{2} }}} x_{1} - \sqrt {\frac{\lambda }{{\tilde{D}_{3} }}} x_{2} }} } \right]\left[ {\left( {\frac{{\tilde{\eta }_{3} }}{{\tilde{\eta }_{4} }}\sqrt {\frac{\lambda }{{\tilde{D}_{3} }}} - \sqrt {\frac{\lambda }{{\tilde{D}_{4} }}} } \right)} \right.e^{{\sqrt {\frac{\lambda }{{\tilde{D}_{4} }}} x_{3} }} - \left( {\frac{{\tilde{\eta }_{3} }}{{\tilde{\eta }_{4} }}\sqrt {\frac{\lambda }{{\tilde{D}_{3} }}} } \right. \hfill \\ \left. {\left. { + \sqrt {\frac{\lambda }{{\tilde{D}_{4} }}} } \right)e^{{ - \sqrt {\frac{\lambda }{{\tilde{D}_{4} }}} x_{3} }} } \right] + \left[ {\left( {\frac{\lambda }{{\sqrt {\tilde{D}_{2} \tilde{D}_{3} } }} - \frac{{\tilde{\eta }_{2} \lambda }}{{\tilde{\eta }_{3} \tilde{D}_{2} }} - \frac{{\tilde{\eta }_{1} }}{{\tilde{\eta }_{2} }}\frac{\lambda }{{\sqrt {\tilde{D}_{1} \tilde{D}_{3} } }} + \frac{{\tilde{\eta }_{1} }}{{\tilde{\eta }_{3} }}\frac{\lambda }{{\sqrt {\tilde{D}_{1} \tilde{D}_{2} } }}} \right)} \right.e^{{\sqrt {\frac{\lambda }{{\tilde{D}_{3} }}} x_{2} - \sqrt {\frac{\lambda }{{\overset{\lower0.5em\hbox{$\smash{\scriptscriptstyle\frown}$}}{D}_{2} }}} x_{1} }} + \left( {\frac{\lambda }{{\sqrt {\tilde{D}_{2} \tilde{D}_{3} } }} + \frac{{\tilde{\eta }_{2} \lambda }}{{\tilde{\eta }_{3} \tilde{D}_{2} }}} \right. \hfill \\ \left. {\left. { + \frac{{\tilde{\eta }_{1} }}{{\tilde{\eta }_{2} }}\frac{\lambda }{{\sqrt {\tilde{D}_{1} \tilde{D}_{3} } }} + \frac{{\tilde{\eta }_{1} }}{{\tilde{\eta }_{3} }}\frac{\lambda }{{\sqrt {\tilde{D}_{1} \tilde{D}_{2} } }}} \right)e^{{\sqrt {\frac{\lambda }{{\tilde{D}_{3} }}} x_{2} + \sqrt {\frac{\lambda }{{\tilde{D}_{2} }}} x_{1} }} } \right]\left[ {\left( {\frac{{\tilde{\eta }_{3} }}{{\tilde{\eta }_{4} }}\sqrt {\frac{\lambda }{{\tilde{D}_{3} }}} - \sqrt {\frac{\lambda }{{\tilde{D}_{4} }}} } \right)e^{{ - \sqrt {\frac{\lambda }{{\tilde{D}_{4} }}} x_{3} }} - \left( {\frac{{\tilde{\eta }_{3} }}{{\tilde{\eta }_{4} }}\sqrt {\frac{\lambda }{{\tilde{D}_{3} }}} + \sqrt {\frac{\lambda }{{\tilde{D}_{4} }}} } \right)e^{{\sqrt {\frac{\lambda }{{\tilde{D}_{4} }}} x_{3} }} } \right] \hfill \\ \end{gathered}$$

Taking radon exhalation from the cover material as the objective function and the cost, thickness of the cover material, and radon concentration on the surface of the cover as the constraints, the radon exhalation control model of the multilayer cover of uranium tailing pile is Eq. (26).

26 $$\begin{gathered} \min J = \tilde{D}_{n + 1} \tilde{a}_{n + 1} \left( {\tilde{w}_{n + 1} - \tilde{p}_{n + 1} } \right) + \tilde{D}_{n + 1} \tilde{p}_{n + 1} \tilde{\eta }_{n + 1} c_{0} - \tilde{v}\tilde{\eta }_{n + 1} c_{0} \hfill \\ s.t\left\{ \begin{gathered} R = {\text{S}} \cdot \sum\limits_{i = 1}^{n} {\tilde{r}_{i} \cdot x_{i} } \le \tilde{P} \hfill \\ \sum\limits_{i = 1}^{n} {x_{i} } \le \tilde{X} \hfill \\ \end{gathered} \right. \hfill \\ \end{gathered}$$

where $\tilde{r}_{i}$ is the integrated cost per unit thickness of material for different covering 1 m^2^ uranium tailings piles. $\tilde{P}$ is the fuzzy total cost of covering material.

### Fuzzy optimization of multilayer coverage radon control

#### Step 1 Coefficient fuzzy

Generally, a membership function can be divided into left and right parts. For a minimization problem, the integral value of the inverse function of the left part reflects the optimistic viewpoint. The integral value of the right part's inverse function reflects the decision maker's pessimistic view. A convex combination of right and left integral values through an index of optimism, called the total integral value, can convert the fuzzy objective values into integral values, and Pareto solutions can be determined based on these integral values.

Let A be a triangular fuzzy number (TFN), denoted by (*a*_1_, *a*_2_, *a*_3_), and its membership function is given by

27 $$\mu_{A} (x) = \left\{ {\begin{array}{*{20}l} {(x - a_{1} )/(a_{2} - a_{1} ),} \hfill & {a_{1} \le x \le a_{2} } \hfill \\ {(x - a_{3} )/(a_{2} - a_{3} ),} \hfill & {a_{2} \le x \le a_{3} } \hfill \\ {0,} \hfill & {otherwise} \hfill \\ \end{array} } \right.$$

where $a_{1}$,$a_{2}$, and $a_{3}$ are real numbers.

Let the left and right membership functions as

28 $$\left\{ \begin{gathered} \mu_{A} (x)^{L} = (x - a_{1} )/(a_{2} - a_{1} ) \hfill \\ \mu_{A} (x)^{R} = (x - a_{3} )/(a_{2} - a_{3} ) \hfill \\ \end{gathered} \right.$$

That is to say, $\alpha$ cut set of $\tilde{A}$ is

$$A\left( \alpha \right) = \left[ {A_{L} \left( \alpha \right),A_{R} \left( \alpha \right)} \right],0 \le \alpha \le 1$$

According to Eq. (25), the triangular fuzzy number of parameters set is $\tilde{A}$, and $\alpha$ is its cut set. Then the radon exhalation fuzzy target left and right functions can be set as Eq. (29) and Eq. (30).

29 $$\begin{aligned} & f\left( {\left[ {\tilde{\beta }} \right]_{\alpha }^{L} ;x_{1} ,x_{2} ,x_{3} } \right) \\ & = {{ - 4\left( \alpha \right)_{\alpha }^{L} \left( {\sqrt {\frac{{\left( {\tilde{D}_{4} } \right)_{\alpha }^{L} }}{{\left( {\tilde{D}_{1} } \right)_{\alpha }^{R} \left( {\tilde{D}_{2} } \right)_{\alpha }^{R} \left( {\tilde{D}_{3} } \right)_{\alpha }^{R} }}} - \frac{1}{{\left( {\tilde{D}_{1} } \right)_{\alpha }^{R} }}\sqrt {\frac{{\left( {\tilde{D}_{4} } \right)_{\alpha }^{L} }}{{\left( {\tilde{D}_{2} } \right)_{\alpha }^{R} }}} } \right)} \mathord{\left/ {\vphantom {{ - 4\left( \alpha \right)_{\alpha }^{L} \left( {\sqrt {\frac{{\left( {\tilde{D}_{4} } \right)_{\alpha }^{L} }}{{\left( {\tilde{D}_{1} } \right)_{\alpha }^{R} \left( {\tilde{D}_{2} } \right)_{\alpha }^{R} \left( {\tilde{D}_{3} } \right)_{\alpha }^{R} }}} - \frac{1}{{\left( {\tilde{D}_{1} } \right)_{\alpha }^{R} }}\sqrt {\frac{{\left( {\tilde{D}_{4} } \right)_{\alpha }^{L} }}{{\left( {\tilde{D}_{2} } \right)_{\alpha }^{R} }}} } \right)} {\left[ {\left( {\frac{\lambda }{{\sqrt {\left( {\tilde{D}_{2} } \right)_{\alpha }^{R} \left( {\tilde{D}_{3} } \right)_{\alpha }^{R} } }} + \frac{{\left( {\tilde{\eta }_{2} } \right)_{\alpha }^{L} \lambda }}{{\left( {\tilde{\eta }_{3} } \right)_{\alpha }^{R} \left( {\tilde{D}_{2} } \right)_{\alpha }^{R} }} - \frac{{\left( {\tilde{\eta }_{1} } \right)_{\alpha }^{L} }}{{\left( {\tilde{\eta }_{2} } \right)_{\alpha }^{R} }}\frac{\lambda }{{\sqrt {\left( {\tilde{D}_{1} } \right)_{\alpha }^{R} \left( {\tilde{D}_{3} } \right)_{\alpha }^{R} } }}} \right.} \right.}}} \right. \kern-\nulldelimiterspace} {\left[ {\left( {\frac{\lambda }{{\sqrt {\left( {\tilde{D}_{2} } \right)_{\alpha }^{R} \left( {\tilde{D}_{3} } \right)_{\alpha }^{R} } }} + \frac{{\left( {\tilde{\eta }_{2} } \right)_{\alpha }^{L} \lambda }}{{\left( {\tilde{\eta }_{3} } \right)_{\alpha }^{R} \left( {\tilde{D}_{2} } \right)_{\alpha }^{R} }} - \frac{{\left( {\tilde{\eta }_{1} } \right)_{\alpha }^{L} }}{{\left( {\tilde{\eta }_{2} } \right)_{\alpha }^{R} }}\frac{\lambda }{{\sqrt {\left( {\tilde{D}_{1} } \right)_{\alpha }^{R} \left( {\tilde{D}_{3} } \right)_{\alpha }^{R} } }}} \right.} \right.}} \\ & \quad \left. { - \frac{{\left( {\tilde{\eta }_{1} } \right)_{\alpha }^{L} }}{{\left( {\tilde{\eta }_{3} } \right)_{\alpha }^{R} }}\frac{\lambda }{{\sqrt {\left( {\tilde{D}_{1} } \right)_{\alpha }^{R} \left( {\tilde{D}_{2} } \right)_{\alpha }^{R} } }}} \right)e^{{ - \sqrt {\frac{\lambda }{{\left( {\tilde{D}_{2} } \right)_{\alpha }^{R} }}} x_{1} - \sqrt {\frac{\lambda }{{\left( {\tilde{D}_{3} } \right)_{\alpha }^{R} }}} x_{2} }} + \left( {\frac{\lambda }{{\sqrt {\left( {\tilde{D}_{2} } \right)_{\alpha }^{R} \left( {\tilde{D}_{3} } \right)_{\alpha }^{R} } }} - \frac{{\left( {\tilde{\eta }_{2} } \right)_{\alpha }^{L} \lambda }}{{\left( {\tilde{\eta }_{3} } \right)_{\alpha }^{R} \left( {\tilde{D}_{2} } \right)_{\alpha }^{R} }} + \frac{{\left( {\tilde{\eta }_{1} } \right)_{\alpha }^{L} }}{{\left( {\tilde{\eta }_{2} } \right)_{\alpha }^{R} }}\frac{\lambda }{{\sqrt {\left( {\tilde{D}_{1} } \right)_{\alpha }^{R} \left( {\tilde{D}_{3} } \right)_{\alpha }^{R} } }}} \right. \\ & \quad \left. {\left. { - \frac{{\left( {\tilde{\eta }_{1} } \right)_{\alpha }^{L} }}{{\left( {\tilde{\eta }_{3} } \right)_{\alpha }^{R} }}\frac{\lambda }{{\sqrt {\left( {\tilde{D}_{1} } \right)_{\alpha }^{R} \left( {\tilde{D}_{2} } \right)_{\alpha }^{R} } }}} \right)e^{{\sqrt {\frac{\lambda }{{\left( {\tilde{D}_{2} } \right)_{\alpha }^{R} }}} x_{1} - \sqrt {\frac{\lambda }{{\left( {\tilde{D}_{3} } \right)_{\alpha }^{R} }}} x_{2} }} } \right]\left[ {\left( {\frac{{\left( {\tilde{\eta }_{3} } \right)_{\alpha }^{L} }}{{\left( {\tilde{\eta }_{4} } \right)_{\alpha }^{R} }}\sqrt {\frac{\lambda }{{\left( {\tilde{D}_{3} } \right)_{\alpha }^{R} }}} - \sqrt {\frac{\lambda }{{\left( {\tilde{D}_{4} } \right)_{\alpha }^{L} }}} } \right)e^{{\sqrt {\frac{\lambda }{{\left( {\tilde{D}_{4} } \right)_{\alpha }^{L} }}} x_{3} }} - \left( {\frac{{\left( {\tilde{\eta }_{3} } \right)_{\alpha }^{L} }}{{\left( {\tilde{\eta }_{4} } \right)_{\alpha }^{R} }}\sqrt {\frac{\lambda }{{\left( {\tilde{D}_{3} } \right)_{\alpha }^{R} }}} } \right.} \right. \\ & \quad \left. {\left. { + \sqrt {\frac{\lambda }{{\left( {\tilde{D}_{4} } \right)_{\alpha }^{L} }}} } \right)e^{{ - \sqrt {\frac{\lambda }{{\left( {\tilde{D}_{4} } \right)_{\alpha }^{R} }}} x_{3} }} } \right] \\ & \quad + \left[ {\left( {\frac{\lambda }{{\sqrt {\left( {\tilde{D}_{2} } \right)_{\alpha }^{L} \left( {\tilde{D}_{3} } \right)_{\alpha }^{L} } }} - \frac{{\left( {\tilde{\eta }_{2} } \right)_{\alpha }^{R} \lambda }}{{\left( {\tilde{\eta }_{3} } \right)_{\alpha }^{L} \left( {\tilde{D}_{2} } \right)_{\alpha }^{L} }} - \frac{{\left( {\tilde{\eta }_{1} } \right)_{\alpha }^{R} }}{{\left( {\tilde{\eta }_{2} } \right)_{\alpha }^{L} }}\frac{\lambda }{{\sqrt {\left( {\tilde{D}_{1} } \right)_{\alpha }^{L} \left( {\tilde{D}_{3} } \right)_{\alpha }^{L} } }} + \frac{{\left( {\tilde{\eta }_{1} } \right)_{\alpha }^{R} }}{{\left( {\tilde{\eta }_{3} } \right)_{\alpha }^{L} }}\frac{\lambda }{{\sqrt {\left( {\tilde{D}_{1} } \right)_{\alpha }^{L} \left( {\tilde{D}_{2} } \right)_{\alpha }^{L} } }}} \right)e^{{\sqrt {\frac{\lambda }{{\left( {\tilde{D}_{3} } \right)_{\alpha }^{L} }}} x_{2} - \sqrt {\frac{\lambda }{{\left( {\tilde{D}_{2} } \right)_{\alpha }^{L} }}} x_{1} }} } \right. \\ & \quad \left. { + \left( {\frac{\lambda }{{\sqrt {\left( {\tilde{D}_{2} } \right)_{\alpha }^{L} \left( {\tilde{D}_{3} } \right)_{\alpha }^{L} } }} + \frac{{\left( {\tilde{\eta }_{2} } \right)_{\alpha }^{R} \lambda }}{{\left( {\tilde{\eta }_{3} } \right)_{\alpha }^{L} \left( {\tilde{D}_{2} } \right)_{\alpha }^{L} }} + \frac{{\left( {\tilde{\eta }_{1} } \right)_{\alpha }^{R} }}{{\left( {\tilde{\eta }_{2} } \right)_{\alpha }^{L} }}\frac{\lambda }{{\sqrt {\left( {\tilde{D}_{1} } \right)_{\alpha }^{L} \left( {\tilde{D}_{3} } \right)_{\alpha }^{L} } }} + \frac{{\left( {\tilde{\eta }_{1} } \right)_{\alpha }^{R} }}{{\left( {\tilde{\eta }_{3} } \right)_{\alpha }^{L} }}\frac{\lambda }{{\sqrt {\left( {\tilde{D}_{1} } \right)_{\alpha }^{L} \left( {\tilde{D}_{2} } \right)_{\alpha }^{L} } }}} \right)e^{{\sqrt {\frac{\lambda }{{\left( {\tilde{D}_{3} } \right)_{\alpha }^{R} }}} x_{2} + \sqrt {\frac{\lambda }{{\left( {\tilde{D}_{2} } \right)_{\alpha }^{R} }}} x_{1} }} } \right] \\ & \quad \left[ {\left( {\frac{{\left( {\tilde{\eta }_{3} } \right)_{\alpha }^{R} }}{{\left( {\tilde{\eta }_{4} } \right)_{\alpha }^{L} }}\sqrt {\frac{\lambda }{{\left( {\tilde{D}_{3} } \right)_{\alpha }^{L} }}} - \sqrt {\frac{\lambda }{{\left( {\tilde{D}_{4} } \right)_{\alpha }^{R} }}} } \right)e^{{ - \sqrt {\frac{\lambda }{{\left( {\tilde{D}_{4} } \right)_{\alpha }^{L} }}} x_{3} }} - \left( {\frac{{\left( {\tilde{\eta }_{3} } \right)_{\alpha }^{L} }}{{\left( {\tilde{\eta }_{4} } \right)_{\alpha }^{R} }}\sqrt {\frac{\lambda }{{\left( {\tilde{D}_{3} } \right)_{\alpha }^{R} }}} + } \right.\left. {\sqrt {\frac{\lambda }{{\left( {\tilde{D}_{4} } \right)_{\alpha }^{R} }}} } \right)e^{{\sqrt {\frac{\lambda }{{\left( {\tilde{D}_{4} } \right)_{\alpha }^{R} }}} x_{3} }} } \right] \\ \end{aligned}$$

30 $$\begin{gathered} f\left( {\left[ {\tilde{\beta }} \right]_{\alpha }^{R} ;x_{1} ,x_{2} ,x_{3} } \right) \hfill \\ = {{ - 4\left( \alpha \right)_{\alpha }^{R} \left( {\sqrt {\frac{{\left( {\tilde{D}_{4} } \right)_{\alpha }^{R} }}{{\left( {\tilde{D}_{1} } \right)_{\alpha }^{L} \left( {\tilde{D}_{2} } \right)_{\alpha }^{L} \left( {\tilde{D}_{3} } \right)_{\alpha }^{L} }}} - \frac{1}{{\left( {\tilde{D}_{1} } \right)_{\alpha }^{L} }}\sqrt {\frac{{\left( {\tilde{D}_{4} } \right)_{\alpha }^{R} }}{{\left( {\tilde{D}_{2} } \right)_{\alpha }^{L} }}} } \right)} \mathord{\left/ {\vphantom {{ - 4\left( \alpha \right)_{\alpha }^{R} \left( {\sqrt {\frac{{\left( {\tilde{D}_{4} } \right)_{\alpha }^{R} }}{{\left( {\tilde{D}_{1} } \right)_{\alpha }^{L} \left( {\tilde{D}_{2} } \right)_{\alpha }^{L} \left( {\tilde{D}_{3} } \right)_{\alpha }^{L} }}} - \frac{1}{{\left( {\tilde{D}_{1} } \right)_{\alpha }^{L} }}\sqrt {\frac{{\left( {\tilde{D}_{4} } \right)_{\alpha }^{R} }}{{\left( {\tilde{D}_{2} } \right)_{\alpha }^{L} }}} } \right)} {\left[ {\left( {\frac{\lambda }{{\sqrt {\left( {\tilde{D}_{2} } \right)_{\alpha }^{L} \left( {\tilde{D}_{3} } \right)_{\alpha }^{L} } }} + \frac{{\left( {\tilde{\eta }_{2} } \right)_{\alpha }^{R} \lambda }}{{\left( {\tilde{\eta }_{3} } \right)_{\alpha }^{L} \left( {\tilde{D}_{2} } \right)_{\alpha }^{L} }} - \frac{{\left( {\tilde{\eta }_{1} } \right)_{\alpha }^{R} }}{{\left( {\tilde{\eta }_{2} } \right)_{\alpha }^{L} }}\frac{\lambda }{{\sqrt {\left( {\tilde{D}_{1} } \right)_{\alpha }^{L} \left( {\tilde{D}_{3} } \right)_{\alpha }^{L} } }}} \right.} \right.}}} \right. \kern-\nulldelimiterspace} {\left[ {\left( {\frac{\lambda }{{\sqrt {\left( {\tilde{D}_{2} } \right)_{\alpha }^{L} \left( {\tilde{D}_{3} } \right)_{\alpha }^{L} } }} + \frac{{\left( {\tilde{\eta }_{2} } \right)_{\alpha }^{R} \lambda }}{{\left( {\tilde{\eta }_{3} } \right)_{\alpha }^{L} \left( {\tilde{D}_{2} } \right)_{\alpha }^{L} }} - \frac{{\left( {\tilde{\eta }_{1} } \right)_{\alpha }^{R} }}{{\left( {\tilde{\eta }_{2} } \right)_{\alpha }^{L} }}\frac{\lambda }{{\sqrt {\left( {\tilde{D}_{1} } \right)_{\alpha }^{L} \left( {\tilde{D}_{3} } \right)_{\alpha }^{L} } }}} \right.} \right.}} \hfill \\ \left. { - \frac{{\left( {\tilde{\eta }_{1} } \right)_{\alpha }^{R} }}{{\left( {\tilde{\eta }_{3} } \right)_{\alpha }^{L} }}\frac{\lambda }{{\sqrt {\left( {\tilde{D}_{1} } \right)_{\alpha }^{L} \left( {\tilde{D}_{2} } \right)_{\alpha }^{L} } }}} \right)e^{{ - \sqrt {\frac{\lambda }{{\left( {\tilde{D}_{2} } \right)_{\alpha }^{L} }}} x_{1} - \sqrt {\frac{\lambda }{{\left( {\tilde{D}_{3} } \right)_{\alpha }^{L} }}} x_{2} }} + \left( {\frac{\lambda }{{\sqrt {\left( {\tilde{D}_{2} } \right)_{\alpha }^{L} \left( {\tilde{D}_{3} } \right)_{\alpha }^{L} } }} - \frac{{\left( {\tilde{\eta }_{2} } \right)_{\alpha }^{R} \lambda }}{{\left( {\tilde{\eta }_{3} } \right)_{\alpha }^{L} \left( {\tilde{D}_{2} } \right)_{\alpha }^{L} }} + \frac{{\left( {\tilde{\eta }_{1} } \right)_{\alpha }^{R} }}{{\left( {\tilde{\eta }_{2} } \right)_{\alpha }^{L} }}\frac{\lambda }{{\sqrt {\left( {\tilde{D}_{1} } \right)_{\alpha }^{L} \left( {\tilde{D}_{3} } \right)_{\alpha }^{L} } }} - } \right. \hfill \\ \left. {\left. {\frac{{\left( {\tilde{\eta }_{1} } \right)_{\alpha }^{R} }}{{\left( {\tilde{\eta }_{3} } \right)_{\alpha }^{L} }}\frac{\lambda }{{\sqrt {\left( {\tilde{D}_{1} } \right)_{\alpha }^{L} \left( {\tilde{D}_{2} } \right)_{\alpha }^{L} } }}} \right)e^{{\sqrt {\frac{\lambda }{{\left( {\tilde{D}_{2} } \right)_{\alpha }^{L} }}} x_{1} - \sqrt {\frac{\lambda }{{\left( {\tilde{D}_{3} } \right)_{\alpha }^{L} }}} x_{2} }} } \right]\left[ {\left( {\frac{{\left( {\tilde{\eta }_{3} } \right)_{\alpha }^{R} }}{{\left( {\tilde{\eta }_{4} } \right)_{\alpha }^{L} }}\sqrt {\frac{\lambda }{{\left( {\tilde{D}_{3} } \right)_{\alpha }^{L} }}} - \sqrt {\frac{\lambda }{{\left( {\tilde{D}_{4} } \right)_{\alpha }^{R} }}} } \right)e^{{\sqrt {\frac{\lambda }{{\left( {\tilde{D}_{4} } \right)_{\alpha }^{R} }}} x_{3} }} - \left( {\frac{{\left( {\tilde{\eta }_{3} } \right)_{\alpha }^{R} }}{{\left( {\tilde{\eta }_{4} } \right)_{\alpha }^{L} }}\sqrt {\frac{\lambda }{{\left( {\tilde{D}_{3} } \right)_{\alpha }^{L} }}} + } \right.} \right. \hfill \\ \left. {\left. {\sqrt {\frac{\lambda }{{\left( {\tilde{D}_{4} } \right)_{\alpha }^{R} }}} } \right)e^{{ - \sqrt {\frac{\lambda }{{\left( {\tilde{D}_{4} } \right)_{\alpha }^{L} }}} x_{3} }} } \right] + \hfill \\ \left[ {\left( {\frac{\lambda }{{\sqrt {\left( {\tilde{D}_{2} } \right)_{\alpha }^{R} \left( {\tilde{D}_{3} } \right)_{\alpha }^{R} } }} - \frac{{\left( {\tilde{\eta }_{2} } \right)_{\alpha }^{L} \lambda }}{{\left( {\tilde{\eta }_{3} } \right)_{\alpha }^{R} \left( {\tilde{D}_{2} } \right)_{\alpha }^{R} }} - \frac{{\left( {\tilde{\eta }_{1} } \right)_{\alpha }^{L} }}{{\left( {\tilde{\eta }_{2} } \right)_{\alpha }^{R} }}\frac{\lambda }{{\sqrt {\left( {\tilde{D}_{1} } \right)_{\alpha }^{R} \left( {\tilde{D}_{3} } \right)_{\alpha }^{R} } }} + \frac{{\left( {\tilde{\eta }_{1} } \right)_{\alpha }^{L} }}{{\left( {\tilde{\eta }_{3} } \right)_{\alpha }^{R} }}\frac{\lambda }{{\sqrt {\left( {\tilde{D}_{1} } \right)_{\alpha }^{R} \left( {\tilde{D}_{2} } \right)_{\alpha }^{R} } }}} \right)} \right.e^{{\sqrt {\frac{\lambda }{{\left( {\tilde{D}_{3} } \right)_{\alpha }^{R} }}} x_{2} - \sqrt {\frac{\lambda }{{\left( {\tilde{D}_{2} } \right)_{\alpha }^{R} }}} x_{1} }} + \hfill \\ \left( {\frac{\lambda }{{\sqrt {\left( {\tilde{D}_{2} } \right)_{\alpha }^{R} \left( {\tilde{D}_{3} } \right)_{\alpha }^{R} } }} + \frac{{\left( {\tilde{\eta }_{2} } \right)_{\alpha }^{L} \lambda }}{{\left( {\tilde{\eta }_{3} } \right)_{\alpha }^{R} \left( {\tilde{D}_{2} } \right)_{\alpha }^{R} }} + \frac{{\left( {\tilde{\eta }_{1} } \right)_{\alpha }^{L} }}{{\left( {\tilde{\eta }_{2} } \right)_{\alpha }^{R} }}\frac{\lambda }{{\sqrt {\left( {\tilde{D}_{1} } \right)_{\alpha }^{R} \left( {\tilde{D}_{3} } \right)_{\alpha }^{R} } }} + } \right.\left. {\left. {\frac{{\left( {\tilde{\eta }_{1} } \right)_{\alpha }^{L} }}{{\left( {\tilde{\eta }_{3} } \right)_{\alpha }^{R} }}\frac{\lambda }{{\sqrt {\left( {\tilde{D}_{1} } \right)_{\alpha }^{R} \left( {\tilde{D}_{2} } \right)_{\alpha }^{R} } }}} \right)e^{{\sqrt {\frac{\lambda }{{\left( {\tilde{D}_{3} } \right)_{\alpha }^{L} }}} x_{2} + \sqrt {\frac{\lambda }{{\left( {\tilde{D}_{2} } \right)_{\alpha }^{L} }}} x_{1} }} } \right] \hfill \\ \left[ {\left( {\frac{{\left( {\tilde{\eta }_{3} } \right)_{\alpha }^{L} }}{{\left( {\tilde{\eta }_{4} } \right)_{\alpha }^{R} }}\sqrt {\frac{\lambda }{{\left( {\tilde{D}_{3} } \right)_{\alpha }^{R} }}} - \sqrt {\frac{\lambda }{{\left( {\tilde{D}_{4} } \right)_{\alpha }^{L} }}} } \right)e^{{ - \sqrt {\frac{\lambda }{{\left( {\tilde{D}_{4} } \right)_{\alpha }^{R} }}} x_{3} }} - \left( {\frac{{\left( {\tilde{\eta }_{3} } \right)_{\alpha }^{R} }}{{\left( {\tilde{\eta }_{4} } \right)_{\alpha }^{L} }}\sqrt {\frac{\lambda }{{\left( {\tilde{D}_{3} } \right)_{\alpha }^{L} }}} + } \right.\left. {\sqrt {\frac{\lambda }{{\left( {\tilde{D}_{4} } \right)_{\alpha }^{L} }}} } \right)e^{{\sqrt {\frac{\lambda }{{\left( {\tilde{D}_{4} } \right)_{\alpha }^{L} }}} x_{3} }} } \right] \hfill \\ \end{gathered}$$

Then the fuzzy objective and fuzzy constraint conversion of the three-layer coverage system is Eq. (31).

31 $$\begin{gathered} \min f\left( {\left[ {\tilde{\beta }} \right]_{\alpha }^{L} ;x_{1} ,x_{2} ,x_{3} } \right) \hfill \\ s.t\left\{ \begin{gathered} \left( {\tilde{P}} \right)_{\alpha }^{L} \le g_{1} \left( {x_{1} ,x_{2} ,x_{3} } \right) \le \left( {\tilde{P}} \right)_{\alpha }^{R} \hfill \\ \left( {\tilde{X}} \right)_{\alpha }^{L} \le g_{2} \left( {x_{1} ,x_{2} ,x_{3} } \right) \le \left( {\tilde{X}} \right)_{\alpha }^{R} \hfill \\ \end{gathered} \right. \hfill \\ \end{gathered}$$

$\tilde{r}_{1} {\kern 1pt} {\kern 1pt} {\kern 1pt} ,{\kern 1pt} {\kern 1pt} {\kern 1pt} {\kern 1pt} {\kern 1pt} \tilde{r}_{2} {\kern 1pt} {\kern 1pt} ,{\kern 1pt} {\kern 1pt} {\kern 1pt} {\kern 1pt} {\kern 1pt} {\kern 1pt} \tilde{r}_{3}$ are the fuzzy composite cost per unit thickness of different covering materials per square meter of uranium tailings pile.$x_{1} ,x_{2} ,x_{3}$ are the thicknesses of different covering materials.

#### Step 2 Objective membership function

Let the extreme value interval of the fuzzy objective function of the three-layer coverage system be Eq. (32).

32 $$\left\{ \begin{gathered} f_{\alpha }^{ - } = \mathop {\min }\limits_{{\left( {Q,x} \right) \in F_{1} }} f\left( {\left[ {\tilde{\beta }} \right]_{\alpha }^{L} ;x_{1} ,x_{2} ,x_{3} } \right) \hfill \\ f_{\alpha }^{ + } = \mathop {\min }\limits_{{\left( {Q,x} \right) \in F_{2} }} f\left( {\left[ {\tilde{\beta }} \right]_{\alpha }^{R} ;x_{1} ,x_{2} ,x_{3} } \right) \hfill \\ \end{gathered} \right.$$

where

33 $$\left\{ \begin{gathered} F_{1} = s.t\left\{ {\tilde{\beta } \ge 0;x_{1} ,x_{2} ,x_{3} \ge 0} \right. \hfill \\ F_{2} = \left\{ {f\left( {\left[ {\tilde{\beta }} \right]_{\alpha }^{L} ;x_{1} ,x_{2} ,x_{3} } \right) \ge f_{\alpha }^{ - } ,x \in F_{1} } \right\} \hfill \\ \end{gathered} \right.$$

Then, the objective membership function is Eq. (33)

34 $$\mu_{{G\left( {\tilde{\beta },x_{1} ,x_{2} ,x_{3} } \right)}} = \left\{ {\begin{array}{*{20}l} 1 \hfill & {f\left( {\left[ {\tilde{\beta }} \right]_{\alpha }^{L} ;x_{1} ,x_{2} ,x_{3} } \right) \le f_{\alpha }^{ - } } \hfill \\ {1 - {{\left( {f\left( {\left[ {\tilde{\beta }} \right]_{\alpha }^{L} ;x_{1} ,x_{2} ,x_{3} } \right) - f_{\alpha }^{ - } } \right)} \mathord{\left/ {\vphantom {{\left( {f\left( {\left[ {\tilde{\beta }} \right]_{\alpha }^{L} ;x_{1} ,x_{2} ,x_{3} } \right) - f_{\alpha }^{ - } } \right)} {\left( {f_{\alpha }^{ + } - f_{\alpha }^{ - } } \right),}}} \right. \kern-0pt} {\left( {f_{\alpha }^{ + } - f_{\alpha }^{ - } } \right),}}} \hfill & {f_{\alpha }^{ - } \prec f\left( {\left[ {\tilde{\beta }} \right]_{\alpha }^{L} ;x_{1} ,x_{2} ,x_{3} } \right) \le f_{\alpha }^{ + } } \hfill \\ 1 \hfill & {f\left( {\left[ {\tilde{\beta }} \right]_{\alpha }^{L} ;x_{1} ,x_{2} ,x_{3} } \right) \succ f_{\alpha }^{ + } } \hfill \\ \end{array} } \right.$$

#### Step 3 Constraint membership function

A profit maximization and cost minimization scheme which considers the existence of possible indeterminacy by designing an uncertain multi-objective multi-item fixed charge solid transportation problem with budget constraint^30^. In the meantime, the unit production cost is a function of production rate and is also dependent on raw material cost, development cost due to reliability, and wear-tear cost^31^. Therefore, the total cost and thickness constraint membership functions are set as Eq. (35) and Eq. (36), respectively.

35 $$\mu_{{C_{1} }} = \left\{ {\begin{array}{*{20}l} 1 \hfill & {g_{1} \left( {x_{1} ,x_{2} ,x_{3} } \right) \le \left( {\tilde{P}} \right)_{\alpha }^{L} } \hfill \\ {{{\left( {g_{1} \left( {x_{1} ,x_{2} ,x_{3} } \right) - \left( {\tilde{P}} \right)_{\alpha }^{L} } \right)} \mathord{\left/ {\vphantom {{\left( {g_{1} \left( {x_{1} ,x_{2} ,x_{3} } \right) - \left( {\tilde{P}} \right)_{\alpha }^{L} } \right)} {\left( {\left( {\tilde{P}} \right)_{\alpha }^{R} - \left( {\tilde{P}} \right)_{\alpha }^{L} } \right),}}} \right. \kern-0pt} {\left( {\left( {\tilde{P}} \right)_{\alpha }^{R} - \left( {\tilde{P}} \right)_{\alpha }^{L} } \right),}}} \hfill & {\left( {\tilde{P}} \right)_{\alpha }^{L} \prec g_{1} \left( {x_{1} ,x_{2} ,x_{3} } \right) \prec \left( {\tilde{P}} \right)_{\alpha }^{R} } \hfill \\ 0 \hfill & {g_{1} \left( {x_{1} ,x_{2} ,x_{3} } \right) \ge \left( {\tilde{P}} \right)_{\alpha }^{R} } \hfill \\ \end{array} } \right.$$

36 $$\mu_{{C_{2} }} \left\{ {\begin{array}{*{20}l} 1 \hfill & {g_{2} \left( {x_{1} ,x_{2} x_{3} } \right) \le \left( {\tilde{X}} \right)_{\alpha }^{L} } \hfill \\ {{{\left( {g_{2} \left( {x_{1} ,x_{2} ,x_{3} } \right) - \left( {\tilde{X}} \right)_{\alpha }^{L} } \right)} \mathord{\left/ {\vphantom {{\left( {g_{2} \left( {x_{1} ,x_{2} ,x_{3} } \right) - \left( {\tilde{X}} \right)_{\alpha }^{L} } \right)} {\left( {\left( {\tilde{X}} \right)_{\alpha }^{R} - \left( {\tilde{X}} \right)_{\alpha }^{L} } \right),}}} \right. \kern-0pt} {\left( {\left( {\tilde{X}} \right)_{\alpha }^{R} - \left( {\tilde{X}} \right)_{\alpha }^{L} } \right),}}} \hfill & {\left( {\tilde{X}} \right)_{\alpha }^{L} \prec g_{2} \left( {x_{1} ,x_{2} ,x_{3} } \right) \prec \left( {\tilde{X}} \right)_{\alpha }^{R} } \hfill \\ 0 \hfill & {g_{2} \left( {x_{1} ,x_{2} ,x_{3} } \right) \ge \left( {\tilde{X}} \right)_{\alpha }^{R} } \hfill \\ \end{array} } \right.$$

#### Step 4 Integration of fuzzy membership functions

①When the fuzzy objective and fuzzy constraint are of equal importance, then its fuzzy set function $\tilde{D}$ can be defined as Eq. (37).

37 $$\mu_{{\tilde{D}}}^{CO} \left( {\tilde{\beta };x_{1} ,x_{2} ,x_{3} } \right) = \min \left( {\mu_{F} \left( {\tilde{\beta };x_{1} ,x_{2} ,x_{3} } \right),\mu_{{C_{1} }} \left( {x_{1} ,x_{2} ,x_{3} } \right),,\mu_{{C_{2} }} \left( {x_{1} ,x_{2} ,x_{3} } \right)} \right)$$

②When the fuzzy objective and fuzzy constraint are of different importance, then its fuzzy set function $\tilde{D}$ can be defined as Eq. (38).

38 $$\begin{gathered} \mu_{{\tilde{D}}}^{CO} \left( {\tilde{\beta };x_{1} ,x_{2} ,x_{3} } \right) = p\mu_{F} \left( {\tilde{\beta };x_{1} ,x_{2} ,x_{3} } \right) + q\mu_{{C_{1} }} \left( {x_{1} ,x_{2} ,x_{3} } \right) + \gamma \mu_{{C_{2} }} \left( {x_{1} ,x_{2} ,x_{3} } \right) \hfill \\ s.t \, p + q + \gamma = 1,p \ge 0,q \ge 0,\gamma \ge 0 \hfill \\ \end{gathered}$$

#### Step 5 Fuzzy swarm intelligence optimization

According to the fuzzy integration maximum membership function law, its objective function is Eq. (39). an interactive procedure was designed to help a decision maker to select a solution from the fuzzy optimal solution^32^. Therefore, the swarm intelligence algorithm is applied to optimize the solution, and the implementation steps are as follows.

39 $$ObjV = \max \mu_{{\tilde{D}}}^{CO} \left( {\tilde{\beta };x_{1} ,x_{2} ,x_{3} } \right)$$

Step 1 Membership function setting. It sets the membership function of fuzzy numbers according to the property of the model parameter coefficient of radon exhalation.

Step 2 Initialization of fuzzy number. The fuzzy number of fuzzy targets and constraints was initialized according to different possible levels.

Step 3 Expected value of the fuzzy objective function. According to the fuzzy parameter initialization of the fuzzy objective, the expected value interval of its corresponding fuzzy objective function is solved under the condition that it meets the corresponding possibility level interval and its constraint conditions.

Step 4 Membership function of fuzzy objective and fuzzy constraint. It constructs the corresponding membership function of fuzzy objective and fuzzy constraint, respectively, according to the expected value interval of fuzzy objective and fuzzy constraint.

Step 5 Fuzzy aggregation function setting. According to the relative importance of fuzzy objectives and constraints, it constructs a fuzzy aggregation function of radon exhalation;

Step 6 Swarm intelligent optimization. The fuzzy aggregation function is solved iteratively by combining the excellent performance of the genetic algorithm, immune algorithm, and particle swarm optimization algorithm.

## Case study

### Example introduction

Based on summarizing the research achievements and development history of uranium tailings (slag) pond covering systems at home and abroad. A large number of covering tests of covering materials for different uranium ore piles, test engineering data of decommissioned covering sites, analysis of covering media, covering site size, compactness, and monitoring methods, and sorting out the coverage parameter range. Take the example of a multi-layer coverage project of uranium tailings in Hunan Province, China. Sandy loam is selected as the first layer of cover material, cement mortar as the second layer of cover material, and asphalt as the third layer of cover material. Given the uncertainty of each correlation parameter, all relevant parameters are described by fuzzy triangular numbers (Table 1). In this example project, the unit coverage cost is calculated based on unit thickness per 100 square meters.

**Table 1:** Fuzzy triangular number of the three-layer coverage parameter coefficients.

Parameter name	parameter symbol	parameter mean	parameter value range			Remark
			**(min)**	**(m)**	**(max)**	
Radon mobility	$\tilde{\alpha }$	2.62	2.42	2.62	2.82	Bq/(m^3^·s)
Decay constant	*λ*	2.1 × 10^−6^	2.1 × 10^−6^	2.1 × 10^−6^	2.1 × 10^−6^	s^−1^
Radon diffusion coefficient of tailings pile	$\tilde{D}_{1}$	3.85 × 10^−6^	3.55 × 10^−6^	3.85 × 10^−6^	4.15 × 10^−6^	m^2^.s^−1^
Porosity of tailings pile	$\tilde{\eta }_{1}$	0.556	0.456	0.556	0.656	
Radon diffusion coefficient of the first covering layer	$\tilde{D}_{2}$	1.63 × 10^−6^	1.43 × 10^−6^	1.63 × 10^−6^	1.83 × 10^−6^	m^2^.s^−1^
Porosity of the first covering layer	$\tilde{\eta }_{2}$	0.23	0.19	0.23	0.27	
Radon diffusion coefficient of the second covering layer	$\tilde{D}_{3}$	0.57 × 10^−6^	0.42 × 10^−6^	0.57 × 10^−6^	0.62 × 10^−6^	m^2^.s^−1^
Porosity of the second covering layer	$\tilde{\eta }_{3}$	0.16	0.13	0.16	0.19	
Radon diffusion coefficient of the third covering layer	$\tilde{D}_{4}$	1.59 × 10^−8^	1.49 × 10^−8^	1.59 × 10^−8^	1.69 × 10^−8^	m^2^.s^−1^
Porosity of the third covering layer	$\tilde{\eta }_{4}$	0.1	0.08	0.1	0.12	
Sandy loam unit price	$\tilde{r}_{1}$	32.700	29.430	32.700	35.970	RMB/m^3^
Cement mortar unit price	$\tilde{r}_{2}$	39.500	35.550	39.500	43.450	RMB/m^3^
Asphalt unit price	$\tilde{r}_{3}$	35.800	32.220	35.800	39.380	RMB/m^3^
Total cost of the covering layer	$\tilde{P}$	35,000	32,000	35,000	37,000	RMB
Total thickness of the covering layer	$\tilde{X}$	1.2	1.0	1.2	1.4	m

### Optimization analysis of radon exhalation

**Figure 3:**
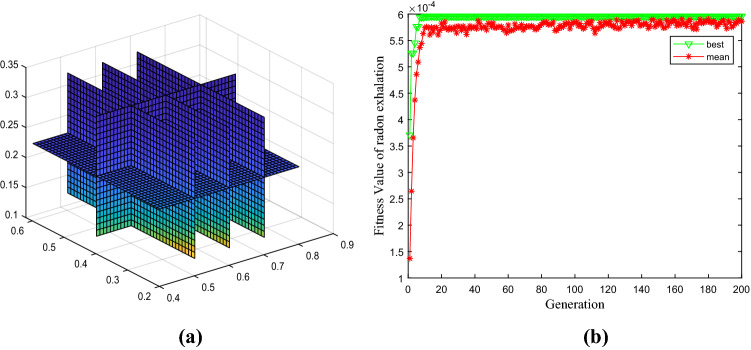
State equation for optimal control of covering material performance parameters. (**a**) Four-dimensional slice diagram of radon exhalation equation. (**b**) Iterative optimization of radon exhalation equation.

The radon control parameters of three-layer coverage are expressed in Eq. (20), and the parameter value adopts the mean value in Table 1. The radon exhalation equation of covering material performance parameters is primary control by the thickness of multilayer covering and relative parameters. The boundary conditions of three-layer coverage are set as lower = c(0.42,0.22,0.12) and upper = c(0.82,0.62,0.32). The optimal solution is obtained by the immune genetic algorithm as[0.4200, 0.2200,0.1200]. The state equation of radon exhalation is shown in Fig. 3, and the optimal fitness objective value is 0.0006.

### Fuzzy optimization analysis of radon exhalation

#### The extreme value interval of the fuzzy target

**Figure 4:**
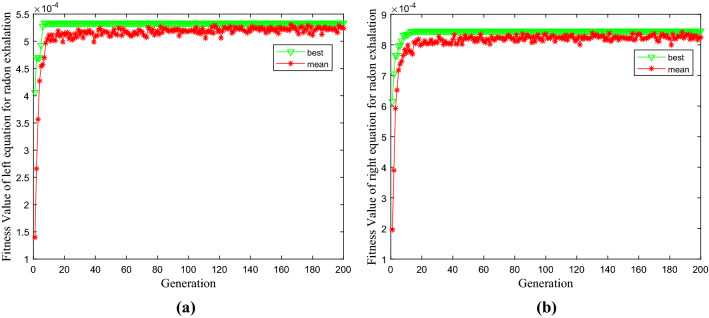
The optimal fitness value of radon exhalation of three-layer coverage. (**a**) the left equation of radon exhalation. (**b**) the right equation of radon exhalation.

The radon control parameters of the three-layer overlay are set as triangular membership functions with their fuzzy tangent sets set to $\alpha$. According to the fuzzy parameter coefficients, the objective function of the left and right equations is constructed for radon exhalation of three-layer coverage. The immune genetic algorithm can be applied to find the extreme value intervals of the left and right target equations, respectively. The optimal fitness value of the left equation is 0.0005 (Fig. 4a), and the optimal solution is [x 1, x 2, x 3] = [0.4200,0.2200,0.1200]. The optimal fitness value of the right equation is 0.0008 (Fig. 4b), and the optimal solution is [x 1, x 2, x 3] = [0.4200,0.2200,0.1200].

#### Integrated analysis of fuzzy target and fuzzy constraint

First, the fuzzy target U_G_ is constructed based on the extreme value interval in Eq. (33). Second, fuzzy constraints U_C1_ and U_C2_ are constructed based on the control standard of radon concentration in Eq. (34) and Eq. (35), respectively. Third, the fuzzy integration function U_GC_ is constructed by combining the importance of fuzzy objectives and fuzzy constraints.

When *p* = *q* = *γ*, the fuzzy integration function is set as

U_GC_ = min([1,ug,uc1,uc2])

**Figure 5:**
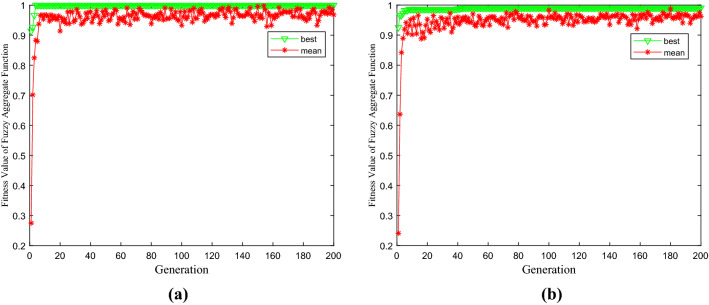
Swarm intelligent optimization of fuzzy integration function of radon exhalation subject to different probability levels and the same important conditions. (**a**) when $\alpha$ = 0.2 $p = q = r$. (**b**) when $\alpha$ = 0.8 $p = q = r$.

According to the different $\alpha$ cut sets of fuzzy parameters, the optimal solution is obtained by the swarm intelligence algorithm with different possibility levels (Table 2). Figure 5 shows that the fuzzy integration function of radon exhalation has a downward trend with the increase of the cut set, but the decline is not obvious. At the same time, this provides a three-layer covering decision database with different possible levels under the same important conditions of fuzzy objectives and fuzzy constraints, and especially there are countless optimal solutions under different cut sets.

**Table 2:** Optimum cover thickness under different possibility levels.

$\alpha$ Cut set	$x_{1}$	$x_{2}$	$x_{3}$	$U_{GC}$
0.1	0.4678	0.2200	0.3111	1.0000
0.2	0.4242	0.2562	0.3022	1.0000
0. 3	0.4532	0.2293	0.3087	1.0000
0.4	0.4624	0.2204	0.3125	1.0000
0.5	0.4202	0.2606	0.3142	1.0000
0.6	0.4267	0.2505	0.3199	1.0000
0.7	0.4201	0.2608	0.3200	0.9977
0.8	0.4220	0.2615	0.3200	0.9911
0.9	0.4223	0.2638	0.3200	0.9847
1.0	0.4226	0.2662	0.3200	0.9782

When fuzzy target and fuzzy constrain are of different importance, the fuzzy integration function is set as

$${\text{U}}_{{{\text{GC}}}} = p*{\text{U}}_{{\text{G}}} + q*{\text{U}}_{{{\text{C}}1}} + \gamma *{\text{U}}_{{{\text{C}}2}}$$

where *p* + *q* + *γ* = 1;

The optimal solution is obtained by the swarm intelligence algorithm with different possibility levels and different importance (Table 3).

**Table 3:** Optimum cover thickness under different possibility and importance levels.

$\alpha$ Cut set	$p$	$q$	$\gamma$	$x_{1}$	$x_{2}$	$x_{3}$	$U_{GC}$
0.2	0.8	0.1	0.1	0.4549	0.2263	0.3163	1.0000
	0.5	0.2	0.3	0.4532	0.2207	0.3096	1.0000
	0.2	0.3	0.5	0.4236	0.2538	0.3167	1.0000
0.5	0.8	0.1	0.1	0.4467	0.2206	0.3183	1.0000
	0.5	0.2	0.3	0.4387	0.2212	0.3183	1.0000
	0.2	0.3	0.5	0.4222	0.2533	0.3199	1.0000
0.8	0.8	0.1	0.1	0.4200	0.2902	0.3200	0.9924
	0.5	0.2	0.3	0.4217	0.2583	0.3200	0.9950
	0.2	0.3	0.5	0.4222	0.2578	0.3200	0.9980

**Figure 6:**
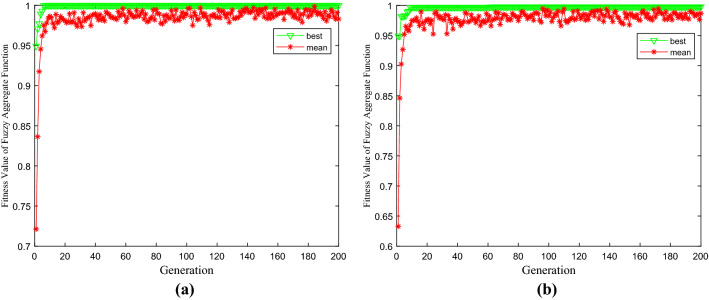
Swarm intelligent optimization of fuzzy integration function of radon exhalation subject to different probability levels and important conditions. (a)when $\alpha$ = 0.5, $p$ = 0.5,$q$ = 0.2, $\gamma$ = 0.3). **(b)** when $\alpha$ = 0.8, $p$ = 0.2,$q$ = 0.3, $\gamma$ = 0.5).

Figure 6 shows that with the increase of the cut set, the fuzzy integration function of radon exhalation has a downward trend. Still, the decline is not apparent, similar to the result of Fig. 5 under the same importance of objectives and constraints. Subject to different probability levels and important conditions such as different fuzzy targets and fuzzy constraints, it obtains the optimal decision-making database of radon exhalation with three-layers coverage by the swarm intelligence algorithm, and there are countless optimal solutions under different cut sets, which provides an essential reference for the construction guidance of multi-layer coverage radon control.

## Conclusion

This study constructed a fuzzy model of the radon control system of uranium tailings under a multilayer coverage mechanism and the optimal decision scheme for the performance parameters of the covering material and the thickness of the multilayer coverage was achieved. The main conclusions are as follows.

### Fuzzy optimization scheme for radon control by multilayer covering

According to the fuzzy parameter coefficients, the objective function of the left and right equations is constructed in regards to the radon exhalation of three-layer coverage. The optimal fitness value of the left and right equation is obtained through immune genetic algorithms, thus providing flexible management for expanding the uncertainty optimization control of radon exhalation.

### Radon control scheme with multilayer cover under different probability levels

The fuzzy aggregation function is reconstructed according to the importance of the fuzzy objective and fuzzy constraint, and the fuzzy integration function of radon exhalation is shown to have a downward trend with the increase of the cut set. At the same time, this provides a three-layer covering decision database with different probability levels under the same importance conditions of fuzzy objectives and constraints.

### Radon control schemes with multilayer covering under different importance levels

Subject to different probability levels and importance conditions, such as different fuzzy targets and fuzzy constraints, this paper obtains the optimal decision-making database of radon exhalation with three-layer coverage using the swarm intelligence algorithm, which guides the construction of multilayer coverage by providing a critical radon control reference.

### Future research plan for radon control by multilayer covering

Through the analysis of three-layer coverage, it is demonstrated that when the dynamic model of radon exhalation is unstable, we should thoroughly discuss the radon exhalation dynamics fuzzy equation, optimize the design of radon control decision variables, build a multi-layer radon control dynamic programming model, and achieve the coordinated optimization of construction quality, cost, progress and radiation safety in uranium tailings covering.

## Data Availability

All data generated or analyzed during this study are included in the manuscript.
